# Identification of a Novel *EVC2* Variant in a Family with Non-Syndromic Tooth Agenesis and Its Potential Functional Implications

**DOI:** 10.3390/genes16111288

**Published:** 2025-10-30

**Authors:** Changqing Yan, Jie Li, Chenying Zhang, Yang Liu, Xiaozhe Wang, Shuguo Zheng

**Affiliations:** Department of Preventive Dentistry, Peking University School and Hospital of Stomatology & National Center for Stomatology & National Clinical Research Center for Oral Diseases & National Engineering Research Center of Oral Biomaterials and Digital Medical Devices & Beijing Key Laboratory of Digital Stomatology & NHC Key Laboratory of Digital Stomatology & NMPA Key Laboratory for Dental Materials, Beijing 100081, China; 2211110614@bjmu.edu.cn (C.Y.); lijie1011@bjmu.edu.cn (J.L.); zhangchy@bjmu.edu.cn (C.Z.); liuyang890@bjmu.edu.cn (Y.L.); kqzsg86@bjmu.edu.cn (S.Z.)

**Keywords:** non-syndromic tooth agenesis, *EVC2*, molecular dynamics simulations, hedgehog signaling, protein–protein interaction, genetic mutation

## Abstract

**Background/Objectives**: Non-syndromic tooth agenesis (NSTA) is a congenital condition that causes the absence of one or more teeth without accompanying systemic abnormalities, which significantly affects quality of life. Genetic factors, including mutations in several specific genes, contribute to the pathogenesis of NSTA. This study investigates a novel *EVC2* mutation in a patient with NSTA and explores its potential pathogenic mechanism, with the aim of enriching the spectrum of pathogenic genes. **Methods**: Whole-exome sequencing (WES) was performed on peripheral blood samples from a patient diagnosed with NSTA. Bioinformatics analysis was utilized to identify the mutation and assess its potential impact on protein structure and function. Molecular dynamics simulations were conducted to analyze structural alterations in the EVC2 protein. The binding affinity between EVC2, EVC, and Smoothened (SMO) was to determine the effect of mutation on protein–protein interaction. Protein localization and expression were analyzed using immunofluorescence and Western blotting. Reverse transcription quantitative PCR (RT-qPCR) was employed to evaluate downstream signaling pathway alterations. **Results**: A novel *EVC2* mutation (c.1657_1660delinsA, p.Glu553_leu554delinsMet) was identified in the proband, and the mutation was maternally inherited. Molecular dynamics simulations revealed that the mutation resulted in a decrease in α-helical content and significant conformational changes in the protein structure. This led to reduced binding affinity between EVC2 and its ligands EVC and SMO, destabilizing the structural integrity of the protein complex. Despite these structural changes, EVC2 protein localization and expression were unaffected. Furthermore, a downregulation of *GLI1* and *SHH* expression was observed, indicating impaired Hedgehog (Hh) signaling. The downregulation of the Hh signaling pathway impairs the tooth development process and may lead to the occurrence of tooth agenesis. **Conclusions**: A novel *EVC2* mutation was identified in a patient with NSTA. Based on molecular dynamics simulations, it is hypothesized that this *EVC2* variant could contribute to the pathogenesis of NSTA by impairing the EVC2-EVC-SMO complex formation, which may lead to downregulation of downstream *GLI1* and *SHH*. These findings provide new insights into the molecular mechanisms underlying *EVC2*-mediated NSTA, suggesting that disruption of Hh signaling may represent a critical pathogenic mechanism.

## 1. Introduction

Non-syndromic tooth agenesis (NSTA) is a congenital condition characterized by the absence of one or more teeth during development, without the presence of other systemic abnormalities [1]. It arises due to disruptions in mesenchymal tissue interactions during the early stages of craniofacial development [2]. NSTA can adversely affect masticatory function and facial aesthetics, leading to a range of physiological and psychological complications that significantly impact the quality of life of affected individuals.

The underlying pathogenesis of NSTA remains incompletely understood, but it is thought to involve a combination of genetic, epigenetic, and environmental factors [3]. Genetic factors play a prominent role in the development of NSTA. To date, several pathogenic genes have been identified, including *EDA*, *PAX9*, *EVC2*, *WNT10A*, *AXIN2*, *WNT10B*, *MSX1*, *LTBP3*, *DSP*, *LRP6*, and *GREM2* [4].

*EVC2*, along with its closely related gene *EVC*, is located on chromosome 4p16. These two genes are arranged in a head-to-head configuration and share a common promoter [5,6]. Mutations in either *EVC2* or *EVC* are known to cause autosomal recessive Ellis-van Creveld syndrome or the autosomal dominant Weyers acrofacial dysostosis [7]. EVC2 is a critical regulator of the Hedgehog (Hh) signaling pathway, which plays a crucial role in various developmental processes [8]. EVC2 is localized within the basal body and the ciliary membrane, where it facilitates the activation of SMO at the primary cilium, initiating Hh signal transduction in vertebrates [9,10,11]. Disruption of the Hh signaling pathway, caused by abnormal expression of key signaling molecules, is associated with tooth developmental defects, including arrested tooth development at the bud stage, which is a hallmark of NSTA [1,12].

In this study, we report a novel *EVC2* mutation (c.1657_1660delinsA, p.Glu553_leu554delinsMet) in a patient diagnosed with NSTA. The pathogenic potential of this mutation was further explored through integrated bioinformatics analysis, molecular dynamics simulations, which additionally validated the expression of tooth development-related genes *SHH* and *GLI1*. Despite these preliminary findings, the precise molecular mechanisms underlying the pathogenicity of this mutation remain to be elucidated in future studies.

## 2. Materials and Methods

### 2.1. Participants

This study was approved by the Ethics Committee of Peking University School and Hospital of Stomatology (Approval Number: PKUSSIRB-202499082) and was conducted in accordance with the Declaration of Helsinki. Written informed consent was obtained from all participants.

The proband was an 11-year-old male who presented with congenital tooth agenesis, without any significant systemic manifestations. Clinical and radiologic examinations confirmed the diagnosis of NSTA. Both parents of the proband underwent clinical and imaging evaluations. The control group consisted of 50 volunteers without congenital tooth agenesis who visited the department for routine dental check-ups.

### 2.2. WES and Bioinformatics Analysis

Genomic DNA was extracted from peripheral blood of the proband and his parents using the TIANamp Genomic DNA Kit (Tiangen, Beijing, China; DP304). DNA libraries were prepared with the SureSelectXT Reagent Kit (Agilent Technologies, Santa Clara, CA, USA; G9611A) and captured using the SureSelect Human All Exon V6 kit (Agilent Technologies; 5190-8863). Sequencing was performed on a HiSeq 2500 system (Illumina, San Diego, CA, USA).

Reads were trimmed and aligned to the human reference genome (GRCh37/hg19) using BWA v0.7.17. Variant calling was performed with SAMtools v1.9 and GATK v4.1, and functional annotation with ANNOVAR (version 20230601). Variants were annotated against dbSNP (build 150) and filtered using population allele-frequency data from the 1000 Genomes Project, ExAC, and the NHLBI ESP6500.

### 2.3. PCR Amplification and Sanger Sequencing

The *EVC2* variant identified by WES was validated by Sanger sequencing. PCR amplification of the exons and exon-intron boundaries of the *EVC2* was performed using intron-exon specific primers (Table 1) as described previously [13]. The PCR reactions were carried out on a DNA Engine PTC-200 (Bio-Rad Laboratories, Hercules, CA, USA). The amplification products were purified and sequenced bidirectionally on an ABI 3730xl DNA Analyzer (Applied Biosystems, Foster City, CA, USA). Sequence data were analyzed using the NCBI BLASTN program (version 2.13.0), and variant nomenclature followed HGVS recommendations.

### 2.4. Prediction of Damaging Effects

Based on the GRCh37/hg19 assembly, the reference genomic DNA and transcript sequences for *EVC2* (Gene ID: 132884; RefSeq: NM_006194.4) were retrieved from the NCBI database. Variant nomenclature and coding consequences were checked with Mutalyzer 3 (https://mutalyzer.nl/). Secondary-structure propensities of the wild-type and mutant proteins were evaluated in Protean (DNASTAR Lasergene 7.1). Three-dimensional homology models were generated on SWISS-MODEL; where applicable, template information and model quality metrics were cross-checked in the SWISS-MODEL Repository. Unless otherwise stated, default parameters were used.

### 2.5. Molecular Dynamics Simulations

#### 2.5.1. System Construction

Molecular models were constructed using AlphaFold3, and molecular dynamics simulations were performed using the Amber22 software. Initial protein conformations were taken from virtual screening. Proteins were parameterized with the ff14SB force field; systems were solvated in TIP3P water using LEaP in a truncated-octahedral box with a 10 Å buffer to the solute. Na^+^ counterions were added to neutralize net charge. Long-range electrostatics were treated with particle-mesh Ewald; a 10 Å cutoff was applied to real-space nonbonded interactions. Periodic boundary conditions were used throughout.

#### 2.5.2. Energy Minimization and Production Dynamics

Energy minimization proceeded in stages with positional restraints as indicated in the original protocol: protein and counterions (200 kcal/mol/Å2), solvent optimization, protein restraints (300 kcal/mol/Å2), and backbone fixation (20 kcal/mol/Å2) to relax side chains (typically 5000 steps steepest descent followed by 5000 steps conjugate gradient per stage). After an unrestrained minimization, systems were heated from 10 K to 300 K over 100 ps under a Langevin thermostat (collision frequency 2/ps) and equilibrated for 500 ps at 1 atm using an isotropic barostat. Production molecular dynamics was run with pmemd.cuda (AMBER 22 GPU implementation) using the SHAKE algorithm on bonds to hydrogen and a 2 fs timestep; coordinates were saved every 10 ps.

#### 2.5.3. Root-Mean-Square Deviation (RMSD) Analysis

Trajectory analyses were performed with cpptraj (AmberTools v22). After least-squares fitting to the minimized starting structure, Cα RMSD was computed; where indicated, receptor-backbone and ligand RMSD were also calculated. Frames from the production runs were sampled every 10 ps. Plots were generated with Grace (xmgrace v5.1.25). An RMSD of 0 Å indicates perfect overlap with the reference frame.

#### 2.5.4. Molecular Mechanics-Generalized Born Surface Area (MM-GBSA) Binding Free Energy

Binding free energies (ΔG_bind) were estimated with the MM-GBSA method implemented in MMPBSA.py (AmberTools v22). Two hundred snapshots were extracted from the stable last 100 ns of the production trajectories (sampling interval, 0.5 ns). Calculations used the GBneck2 model (igb = 8, mbondi3 radii) with an ionic strength of 0.150 M. ΔG_bind was decomposed into molecular mechanics and solvation terms (ΔE_MM + ΔG_solv). Normal-mode entropic contributions were not included owing to computational cost. Procedures followed the AMBER Reference Manual (v22).

### 2.6. Plasmid Construction and Cell Transfection

The *EVC2* cDNA (GenBank NM_006194.4) was cloned into the pcDNA3.1(+)-EGFP vector to generate the wild-type construct. The mutant *EVC2* construct carrying the specified substitution was synthesized by Tsingke Biotechnology (Beijing, China) and sequence-verified.

For transfection, HEK-293T cells (BeNa Culture Collection, Beijing, China) were cultured in DMEM supplemented with 10% FBS (Cyagen, Guangzhou, China; FBSAD-01011-500) at 37 °C in a humidified incubator with 5% CO_2_. Cells were seeded in 6-well plates at 5 × 10^5^ cells/well and allowed to attach for 24 h. Plasmids (wild-type *EVC2*, mutant *EVC2*, or empty vector) were transfected using Lipofectamine 3000 (Thermo Fisher Scientific, Waltham, MA, USA; L3000008) according to the manufacturer’s protocol. Briefly, 2 µg plasmid DNA was mixed with 5 µL Lipofectamine 3000 in Opti-MEM Reduced-Serum Medium (Thermo Fisher Scientific; 31985-070) and incubated for 20 min at room temperature to form lipid-DNA complexes, which were then added to cells in 2 mL DMEM/10% FBS. After 48 h, cells were harvested for downstream assays (subcellular localization and Western blotting).

### 2.7. Subcellular Localization

HEK-293T cells were transfected with wild-type *EVC2*, mutant *EVC2*, or an empty vector and cultured for 48 h. Cells were fixed with 4% paraformaldehyde (Solarbio, Beijing, China; P1110) for 10 min at room temperature, then permeabilized with 0.3% Triton X-100 (Solarbio) in PBS for 5 min. Nuclei were counterstained with DAPI (Abcam, Cambridge, UK; ab104139) at 1 µg/mL for 5 min and washed twice in PBS. Fluorescence images were acquired on an Olympus fluorescence microscope (Olympus Corporation, Tokyo, Japan) controlled by cellSens software v4.2 with appropriate filters for DAPI and EGFP. Protein localization was assessed by comparing EGFP signals from wild-type and mutant EVC2 with nuclear DAPI staining.

### 2.8. Western Blot

Cells were lysed in RIPA buffer (Huaxingbio, Beijing, China; HX1862-2) supplemented with 50× protease inhibitor cocktail (Huaxingbio, China; HX1863). Protein concentrations were determined using the Pierce BCA Protein Assay Kit (Thermo Scientific; A55864). Equal amounts of protein (30 µg) were separated by 4–12% gradient gel and transferred to PVDF membranes (Immobilon-P, 0.45 µm; Merck Millipore, Burlington, MA, USA; IPVH00010) using a semi-dry transfer (100 V, 1 h). Membranes were blocked with 5% (*w*/*v*) non-fat milk in TBST for 1 h at room temperature and incubated overnight at 4 °C with anti-EVC2 primary antibody (Proteintech, Rosemont, IL, USA; 55367-1-AP; 1:1000). After washing, membranes were incubated with HRP-conjugated goat anti-rabbit secondary antibody for 1 h at room temperature (Proteintech; SA00001-2). Protein bands were detected using ECL substrate (NCM Biotech, Suzhou, China; P10300) and imaged on a Fusion FX system (Vilber, Collégien, France). Band intensities were quantified in ImageJ v1.53 (National Institutes of Health, Bethesda, MD, USA).

### 2.9. RNA Extraction and RT-qPCR

Total RNA was isolated from peripheral blood collected from both patients and healthy controls using the PAXgene Blood RNA Kit (Qiagen, Hombrechtikon, Switzerland; 762164). cDNA was synthesized from 500 ng of total RNA using the PrimeScript RT Reagent Kit (Takara, Shiga, Japan; RR037Q). qPCR was performed with FastStart Universal SYBR Green Master (ROX) (Roche, Basel, Switzerland; 4913914001) on a 7500 Real-Time PCR System (Applied Biosystems, Thermo Fisher Scientific) under standard cycling conditions. Relative gene expression was quantified using the ΔΔCt method, with *GAPDH* used as the internal reference gene. Primer sequences are provided in Table 2.

### 2.10. Statistical Analysis

Data are presented as mean ± standard deviation (SD). All experiments were performed in triplicate. Statistical significance was determined using a two-tailed Student’s *t*-test. Statistical significance was considered at *p* < 0.05. All statistical analyses were performed using GraphPad Prism 8.0.2 (GraphPad, San Diego, CA, USA).

## 3. Results

### 3.1. Clinical Manifestations

**Figure 1:**
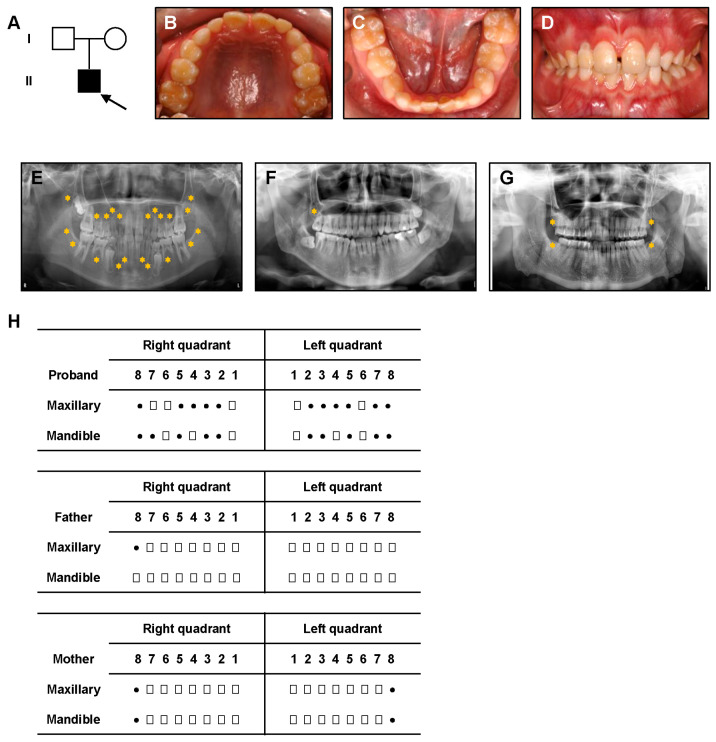
Clinical Presentation of the NSTA Proband. (**A**) Family pedigree of the NSTA proband (indicated by an arrow). (**B**–**D**) Intraoral photographs of the proband, showing no significant dental abnormalities. (**E**–**G**) Panoramic radiographs of the proband (**E**), the proband’s father (**F**), and the proband’s mother (**G**). The yellow stars indicate the absence of permanent tooth buds. (**H**) Diagram illustrating the position of missing teeth. Squares represent erupted permanent teeth, while dots indicate missing permanent tooth germs.

The family pedigree of the proband is shown in Figure 1A, with the proband indicated by an arrow. The proband is an 11-year-old boy in the mixed dentition stage. Clinical examination revealed no significant soft tissue or dental anomalies, as seen in the intraoral photographs (Figure 1B–D).

Panoramic radiographs showed the absence of 17 permanent teeth in the proband, excluding the third molars (Figure 1E). This extensive agenesis was observed in both the maxilla and mandible. The proband underwent a thorough systemic examination at a general hospital to rule out potential syndromic conditions. Physical examination revealed no abnormalities of the extremities or joints (Figure S1). Echocardiography showed normal cardiac structure and function (Figure S2). Based on the oral manifestations and absence of systemic abnormalities, the proband was diagnosed with NSTA. Clinical data were also obtained from the parents. Both parents’ intraoral examinations and radiographs showed no missing teeth except the third molar (Figure 1F,G, Figure S3 and S4). Physical examination of the parents revealed no significant abnormalities (Figure S4). The clinical characteristics of the proband and his parents are summarized in Table 3.

### 3.2. A Novel EVC2 Mutation Identified by WES and Sanger Sequencing

**Figure 2:**
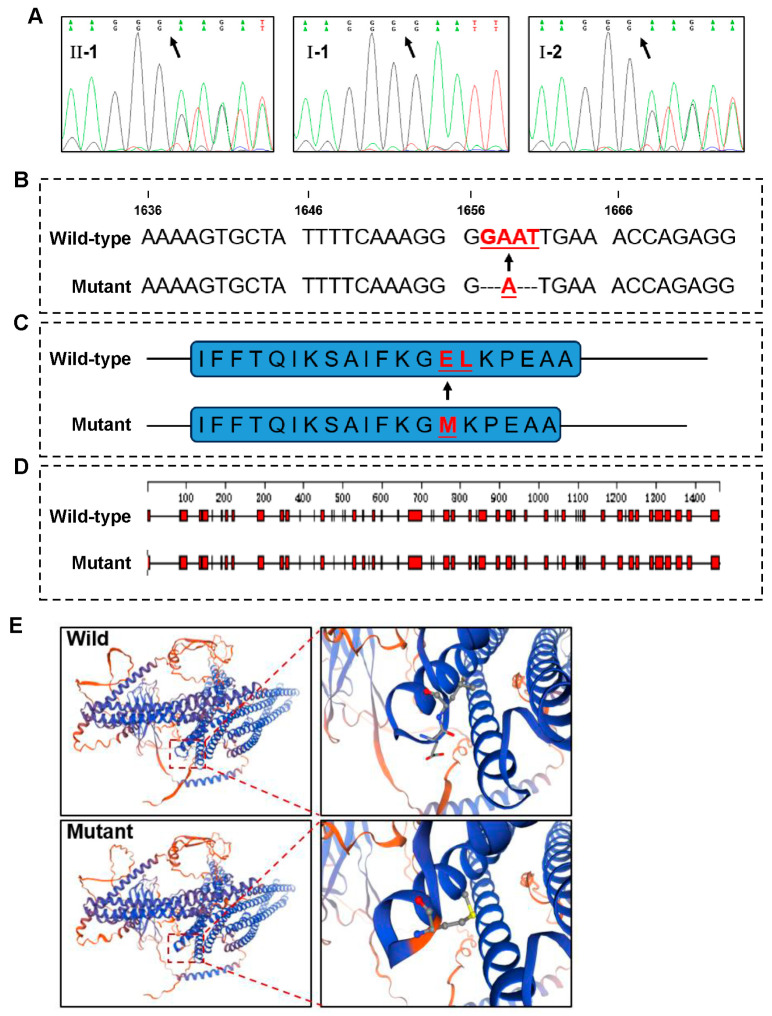
Validation of Mutation Sites and Bioinformatics Analysis. (**A**) Sanger sequencing results of the proband and his parents. (**B**) Schematic diagram of the base change between wild-type and mutant *EVC2*. (**C**) Schematic diagram of the corresponding amino acid change. (**D**) Secondary structure changes in the EVC2 protein due to the mutation. (**E**) Three-dimensional model of the wild-type and mutant proteins, with emphasis on structural changes at the mutation site.

WES analysis identified a novel mutation (c.1657_1660delinsA, p. Glu553_leu554delinsMet) in exon 11 of the *EVC2* gene, which is associated with congenital tooth agenesis. This mutation was confirmed by Sanger sequencing, which showed that both the proband (II-1) and his mother (I-2) carried the same mutation, while the father (I-1) did not harbor the mutation (Figure 2A). Importantly, the mother of the proband did not exhibit any missing teeth, apart from the third molars, indicating the mutation may have incomplete penetrance in some individuals.

### 3.3. Potential Deleterious Effects of the EVC2 Mutation

The identified mutation (c.1657_1660delinsA) results in a base change that leads to a frameshift at the corresponding codon (Figure 2B), causing an amino acid alteration from Glu553_Leu554delinsMet (Figure 2C). Bioinformatics analysis indicated that the mutation could result in a significant loss of the alpha-helical content of the EVC2 protein (Figure 2D).

Structural analysis of the wild-type and mutant EVC2 proteins through three-dimensional modeling further confirmed the potential impact of the mutation on protein structure. The mutant protein exhibited noticeable conformational changes, particularly around the mutation site, where the mutation caused a disruption in the protein’s secondary structure (Figure 2E). The wild-type protein maintains a stable structure, but the mutant version demonstrates significant structural reorganization, likely impairing its functional capacity. These structural changes may contribute to the abnormal tooth development seen in the proband, providing insights into the molecular basis of NSTA.

### 3.4. Potential Impact of EVC2 Mutation on Protein Stability

#### 3.4.1. EVC2 and EVC Complex

**Figure 3:**
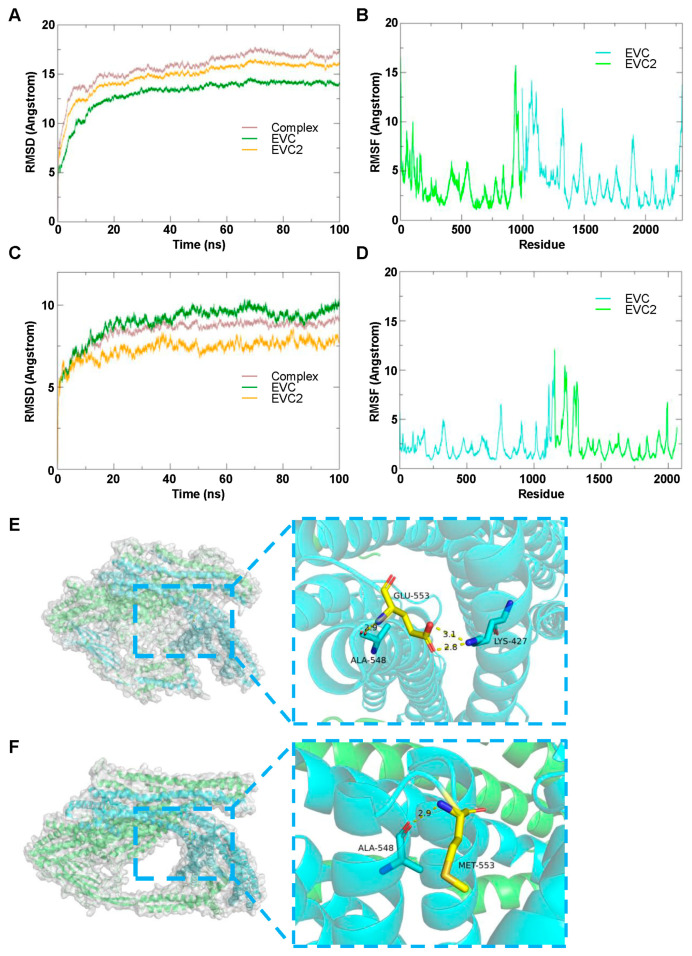
Molecular dynamics simulation of wild-type and mutant EVC2 binding to EVC. (**A**) RMSD changes in the wild-type EVC2/EVC complex during the simulation. RMSD is used to assess the global structural stability of protein conformations. (**B**) RMSF changes in the wild-type EVC2/EVC complex across residues. RMSF, in contrast, is employed to analyze the local flexible fluctuations of specific residues in proteins. (**C**) RMSD changes in the mutant EVC2/EVC complex during the simulation. (**D**) RMSF changes in the mutant EVC2/EVC complex across residues. (**E**) Visual analysis of the wild-type EVC2/EVC binding. (**F**) Visual analysis of the mutant EVC2/EVC binding. In both (**E**,**F**), EVC2 is represented in indigo, EVC in green, and hydrogen bonds are shown as yellow dashed lines. Key residues, including Ala-548, Glu-553, Lys-427, and Met-553, are highlighted in the zoomed-in sections. Collectively, these results suggest that the *EVC2* mutation may alter the conformational dynamics and stability of the EVC2-EVC complex.

Molecular dynamics simulations were performed to evaluate the stability of the wild-type and mutant EVC2/EVC complexes. The RMSD analysis of the wild-type EVC2/EVC complex (Figure 3A) and the mutant EVC2/EVC complex (Figure 3C) showed that both systems maintained stable fluctuations over time. After 20 ns of simulation, both complexes consistently exhibited RMSD values within a low range of approximately 3 Å, indicating high structural stability of the EVC2/EVC system, regardless of the mutation.

The root-mean-square fluctuation (RMSF) analysis further demonstrated that, apart from a slight increase in RMSF at the C-terminal and N-terminal regions (Figure 3B,D), the majority of the amino acid residues in the systems exhibited low RMSF values, suggesting minimal fluctuations and stable protein dynamics. These results indicate that the mutation does not significantly affect the overall stability of the complex.

Visualization of the average conformations of the EVC2/EVC complex (Figure 3E) and the post-mutation EVC2/EVC complex (Figure 3F) was conducted using ten frames taken from the stable state of each simulation. In the wild-type EVC2/EVC system (Figure 3E), the key residue Glu-553 formed three hydrogen bonds with Ala-548 and Lys-427, which stabilizes the structure. In contrast, after the mutation of Glu-553 to Met, only one hydrogen bond with Ala-548 remained (Figure 3F). This indicates that the wild-type Glu-553 contributes more significantly to the stability of the surrounding amino acids compared to the mutant Met-553.

#### 3.4.2. EVC2, EVC and SMO Complex

**Figure 4:**
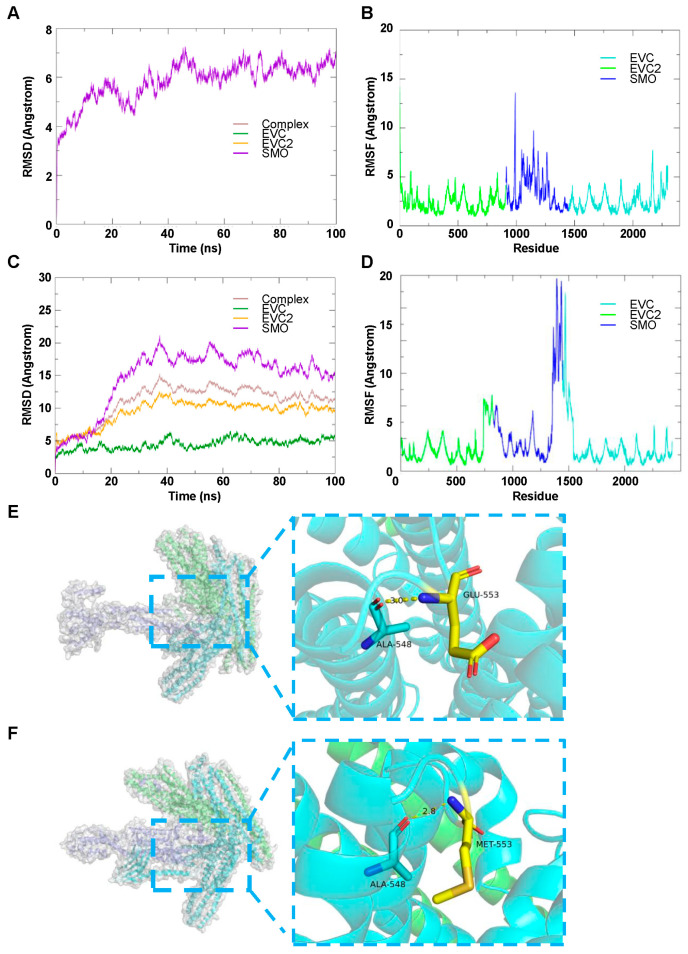
Molecular dynamics simulation of wild-type and mutant EVC2/EVC binding to SMO. (**A**) RMSD changes in the wild-type EVC2/EVC/SMO complex during the simulation. (**B**) RMSF changes in the wild-type EVC2/EVC/SMO complex across residues. (**C**) RMSD changes in the mutant EVC2/EVC/SMO complex during the simulation. (**D**) RMSF changes in the mutant EVC2/EVC/SMO complex across residues. (**E**) Visual analysis of the wild-type EVC2/EVC/SMO binding, with EVC2 represented in indigo, EVC in green, and SMO in blue. Hydrogen bonds are shown as yellow dashed lines, and key residues, including Ala-548 and Glu-553, are highlighted in the zoomed-in section. (**F**) Visual analysis of the mutant EVC2/EVC/SMO binding, with EVC2 represented in indigo, EVC in green, and SMO in blue. Hydrogen bonds are shown as yellow dashed lines, and key residues, including Ala-548 and Glu-553, are highlighted in the zoomed-in section. Molecular dynamics results suggest that the *EVC2* mutation may reduce the conformational stability of the EVC2-EVC protein complex, potentially weakening its interaction with SMO.

To investigate the molecular dynamics of wild-type and mutant EVC2/EVC binding to SMO, we conducted extensive simulations to evaluate the stability and flexibility of the complexes over time. The RMSD analysis of the wild-type EVC2/EVC/SMO complex revealed a gradual increase in RMSD, reaching a plateau after approximately 50,000 ps (Figure 4A). This suggests that the complex achieved an equilibrium state during the simulation. In contrast, the RMSD of the mutant complex showed a more pronounced fluctuation, indicating a less stable binding interaction between EVC2/EVC and SMO (Figure 4C).

To further assess the flexibility of individual residues within the complexes, the RMSF values were calculated across all residues. The wild-type complex exhibited minimal RMSF variation, particularly in the core regions of the EVC2/EVC binding interface, indicating relatively stable interactions (Figure 4B). However, the RMSF values for the mutant complex were notably higher, particularly around the interface residues of EVC2 and SMO, suggesting increased flexibility and weaker interactions in the mutant form (Figure 4D).

In-depth visual analysis of the binding interactions revealed distinct differences between the wild-type and mutant complexes. The wild-type complex showed stable hydrogen bond interactions between key residues, including Ala-548 and Glu-553, which were maintained throughout the simulation (Figure 4E). These residues are crucial for stabilizing the EVC2/EVC/SMO interface. In the mutant complex, the hydrogen bond between Ala-548 and Glu-553 was disrupted, and a new hydrogen bond was formed with Met-553 instead, leading to a different binding configuration (Figure 4F). These structural changes suggest that the mutation may impair the proper alignment of the binding interface, weakening the interaction between the proteins. These results provide valuable insights into the molecular mechanisms underlying the stability and functionality of the EVC2/EVC/SMO complex and highlight the potential effects of mutations on protein–protein interaction.

#### 3.4.3. MM-GBSA Analysis

MM-GBSA analysis was conducted to calculate the total binding free energy (ΔG_total_) between the wild-type (wild-type-EVC2/EVC) and mutant (mutant-EVC2/EVC) complexes. The total binding free energy was broken down into individual components: electrostatic energy (ΔG_ele_), van der Waals energy (ΔG_vdw_), and solute-solvent interaction energies, including polar (ΔG_polar_) and non-polar contributions (ΔG_nonpolar_) (Table 4).

The results revealed that the ΔG_total_ for the wild-type-EVC2/EVC complex (−60,888.0 kcal/mol) was lower than that for the mutant-EVC2/EVC complex (−56,411.4 kcal/mol), indicating a higher binding affinity of the wild-type-EVC2 for EVC. Among the individual components, the electrostatic interactions were the major contributors to the total binding energy in both the wild-type and mutant complexes, with values of ΔG_ele_ = −81,154.6 kcal/mol and ΔG_ele_ = −71,398.6 kcal/mol, respectively.

After mutation, we observed a decrease in the van der Waals (ΔG_vdw_) and nonpolar (ΔG_nonpolar_) energy contributions, while the polar energy (ΔG_polar_) became less favorable. These results highlight the distinct differences in binding affinity between the wild-type and mutant complexes, suggesting that the mutation affects key molecular interactions. Statistical analysis of the *p*-values (all *p* < 0.01) in Table 4 further supports the conclusion that the binding energies of the two complexes differ significantly across all components. Binding free energy calculations, which further validated the molecular docking, revealed that the *EVC2* mutation significantly weakens the binding affinity between EVC2 and EVC. This result demonstrates a noticeable destabilizing effect on the protein complex. However, due to the computational complexity and potential inaccuracies associated with modeling the large-scale EVC2-EVC-SMO complex, further analysis of this system was not pursued.

### 3.5. Impact of EVC2 Mutations on Subcellular Localization

**Figure 5:**
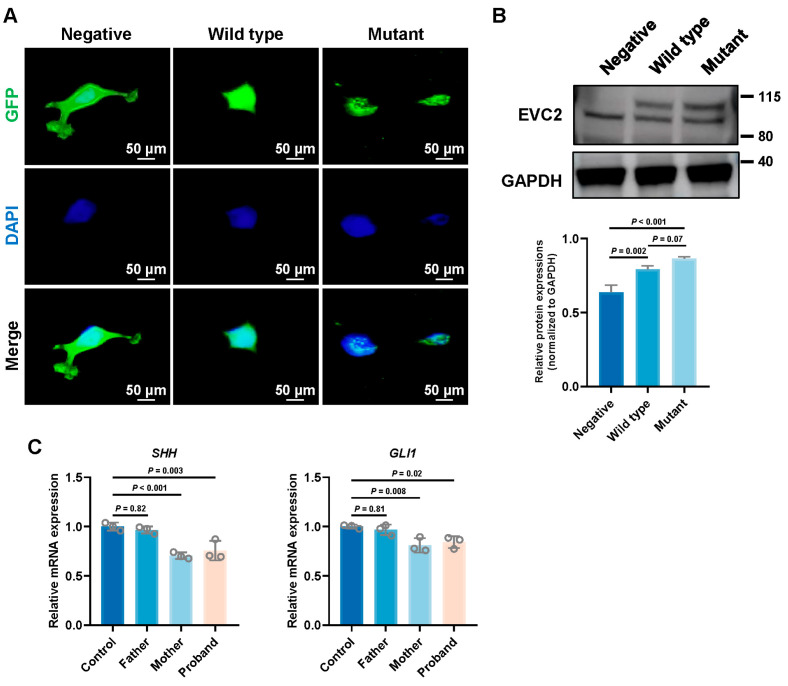
Results of functional analysis of the *EVC2* variant. (**A**) Immunofluorescence assay showing the nuclear localization of both wild-type and p.Glu553_leu554delinsMet mutant EVC2 proteins. The empty vector serves as the negative control. (**B**) Western blot analysis demonstrating that the overexpression of wild-type and mutant EVC2 does not significantly affect protein expression. (**C**) RT-qPCR analysis using cDNA derived from peripheral blood revealed that the *EVC2* mutation significantly altered the expression levels of *GLI1* and *SHH*. Data are presented as mean ± SD (*n* = 3). Statistical significance in (**B**,**C**) was determined by a two-tailed unpaired *t*-test.

Using fluorescence microscopy (Olympus, Japan; IX53), we observed that both the wild-type and the p.Glu553_leu554delinsMet mutant EVC2 proteins localized to the nucleus. The distribution of the mutant EVC2 protein was similar to that of the wild-type, indicating that the p.Glu553_leu554delinsMet mutation did not alter the subcellular localization of EVC2 (Figure 5A).

### 3.6. Impact of EVC2 Mutation on Hedgehog Signaling Pathway

To evaluate the effect of *EVC2* mutations on protein expression, we performed Western blot analysis. Both wild-type and mutant EVC2 proteins were overexpressed, but no significant difference in protein expression was observed between the wild-type and mutant *EVC2* groups (Figure 5B). This suggests that the p.Glu553_leu554delinsMet mutation does not significantly affect EVC2 protein expression.

Next, to investigate the functional consequences of the p.Glu553_leu554delinsMet mutation, we analyzed the expression of *GLI1* and *SHH*, key downstream targets of the Hh signaling pathway that are crucial for tooth development [14]. RT-qPCR results revealed a significant reduction in the expression levels of *GLI1* and *SHH* in the proband and his mother compared to normal controls (Figure 5C). This finding indicates an association between the *EVC2* variant and altered Hh pathway activity. However, the fact that the clinically unaffected mother exhibits a similar molecular profile suggests that this downregulation may be necessary but not sufficient to cause the tooth agenesis, or that it represents a secondary effect within a broader dysregulated network. Therefore, while these data implicate Hh signaling dysregulation in these mutation carriers, the direct mechanistic link to the patient’s specific phenotype requires further investigation.

## 4. Discussion

To date, more than a dozen genes have been clearly linked to NSTA, although a substantial number of associated pathogenic genes and mutation sites remain unidentified. The clinical relationship between *EVC2* and tooth agenesis is increasingly supported, as observed not only in patients with syndromic forms but also in individuals with NSTA [6,15,16,17,18,19,20,21] as summarized in Table 5. While *EVC2* has emerged as a promising candidate gene for congenital tooth agenesis, the exact pathogenic mechanisms remain elusive. In this study, we identified and validated a novel *EVC2* mutation through WES and Sanger sequencing, suggesting its potential role as a causative gene for NSTA. Notably, we utilized molecular dynamics simulations to explore the pathogenic mechanisms linking *EVC2* to NSTA, focusing on the stability of the EVC2/EVC complex and its interactions with other proteins.

*EVC2* spans approximately 150 kb of the human genome, consisting of 22 exons and encoding a 1308-amino acid protein. This protein includes key functional domains, such as the EVC domain, cilia localization domain, and protein complex interaction domain [9,22]. Mutations in *EVC2* can lead to Ellis-van Creveld syndrome or Weyers acrofacial dysostosis, both of which are commonly associated with dental developmental defects. *EVC* and *EVC2* encode single-pass transmembrane proteins that form a heterodimeric complex, essential for maintaining protein stability [14]. This complex is localized within the primary cilia, where it plays a critical role in the Hh signaling pathway [15].

To investigate the potential effect of *EVC2* mutation on protein stability and function, molecular dynamics simulations were employed. These simulations focused on assessing the structural integrity of the EVC2/EVC complex, which is essential for maintaining proper protein interactions. The mutation caused a reduction in hydrogen bonding, decreasing from three bonds (originally formed by Ala548 and Lys427) to only one bond retained by Ala548. Hydrogen bonds are essential for maintaining protein secondary structures, including α-helices and β-sheets [23]. The substitution of glutamic acid with methionine resulted in significant structural changes: the side chain length decreased, charge distribution on the protein surface altered, and hydrogen bonds with surrounding residues were disrupted. These changes appeared to caused limited backbone movement and triggered conformational shifts, potentially destabilizing the protein structure [24]. Based on these computational observations, we hypothesize that the structural instability of EVC2 may impair its interaction with the EVC protein, possibly contributing to disruption of Hh signaling.

We further contextualized these structural predictions within the established biology of Hh signaling. The EVC/EVC2 complex is known to be essential for the precise transduction of Hh signals [10], which act as critical morphogens during tooth development [25]. The conformational flexibility and interaction interfaces affected by the mutation are precisely those required for the complex’s proper function at the primary cilium. Therefore, we hypothesize that the computed structural destabilization may not merely alter static protein properties but could specifically compromise the dynamic protein–protein interaction necessary for effective Hh signal transduction. This hypothesized mechanism provides a functional link between the mutation and the observed phenotype.

Hh signaling, transduced by SMO, is essential for various developmental processes, including tooth development [26,27]. EVC/EVC2 are known to transduce Hh signaling downstream of SMO activation by promoting GLI activation and antagonizing Sufu [10]. Our molecular docking simulations with SMO suggested that the *EVC2* mutation weakened the interaction between EVC2, EVC, and SMO, resulting in altered conformational states. Although the *EVC2* mutation did not significantly affect its protein expression, the resulting structural alterations could potentially compromise interactions with essential ligands, suggesting a possible pathogenic mechanism.

Furthermore, Hh signaling is critical for tooth germ formation and development [15,28]. Binding of SHH to the PTCH1 receptor triggers a downstream cascade that culminates in GLI1 accumulation and activation of target-gene expression [29,30]. In the present study, reduced expression of *GLI1* and *SHH* was observed in both the proband and his mother, indicating a possible association between the *EVC2* variant and altered Hh pathway activity. Considering the mother’s normal phenotype, the functional implications of this observed reduction remain to be fully elucidated. Nevertheless, this molecular profile is compatible with a potential role for Hh signaling attenuation in the pathogenesis of NSTA, warranting further investigation.

The association between *EVC2* and congenital tooth agenesis has been increasingly recognized, in both Ellis-van Creveld syndrome and NSTA [17,31]. In this study, we identified a previously unreported heterozygous *EVC2* mutation in a patient with NSTA, associated with the congenital absence of 17 permanent tooth germs, in the absence of other syndromic features. The variant was maternally inherited, yet the mother had no clinical evidence of tooth agenesis, demonstrating genotype-phenotype discordance despite a shared genotype. Such discordance is frequently observedin genetic disorders and is most often attributed to incomplete penetrance and/or variable expressivity [32,33,34,35]. Perveen et al. identified the same PITX2 mutation in a mother with Axenfeld-Rieger syndrome and her two daughters. Only the mother and one daughter exhibited dental anomalies, suggesting the existence of a compensatory mechanism despite shared PITX2 haploinsufficiency [30]. The manifestation of a genetic variant can be modulated by a spectrum of factors, including environmental influences and polygenic factors, such as the action of genetic modifiers in other loci [36,37,38]. Furthermore, the concept of “transcriptotype”—referring to the precise spatiotemporal expression pattern of an allele—has emerged as another potential contributor to incomplete penetrance and variable expressivity in human disease [39]. In our case, the divergent phenotypes between the proband and the mother may reflect the influence of modifier genes and/or environmental factors. Advances in detection technologies provide valuable opportunities for deeper investigation into the pathogenic mechanisms underlying NSTA [40].

This study has several limitations that should be acknowledged. First, the functional analyses were conducted in HEK-293T cells, which do not fully recapitulate the molecular microenvironment of tooth development. Consequently, the observed Hh pathway downregulation, while providing valuable insights, may not accurately reflect the situation in native odontogenic cells. Future work should validate these findings in more relevant models, such as dental pulp stem cells or odontoblast-like cells. Furthermore, while informative, molecular dynamics simulations alone cannot fully establish the pathogenic role of the *EVC2* variant in NSTA. Ultimately, the generation and analysis of an *Evc2* knockout mouse model will be invaluable for definitively establishing the in vivo pathogenic mechanism and understanding its role in tooth development.

## Figures and Tables

**Table 1:** Primers used for DNA amplification of *EVC2*.

Primer	Primer Sequence (5′-3′)	Location	Size of Amplified Fragment (bp)
Forward	CAACCTTCTGCGGACCCT	Exon 11	380
Reverse	TCCCTGCTTACTGGAAACTCAC	Exon 11	380

**Table 2:** Primers for RT-qPCR.

Genes	Primer	Primer Sequence (5′-3′)
*GLI1*	Forward	AGCGTGAGCCTGAATCTGTG
	Reverse	CAGCATGTACTGGGCTTTGAA
*SHH*	Forward	CTCGCTGCTGGTATGCTCG
	Reverse	ATCGCTCGGAGTTTCTGGAGA
*GAPDH*	Forward	GTCTCCTCTGACTTCAACAGCG
	Reverse	ACCACCCTGTTGCTGTAGCCAA

**Table 3:** Clinical and genetic characteristics of the proband and parents.

Subject	Genotype	Phenotype
Proband	c.1657_1660delinsA	NSTA; absence of 17 permanent teeth
Father	Wild-type	Absence of 1 third molar (within normal variation)
Mother	c.1657_1660delinsA	No clinical anomalies; absence of third molars (within normal variation)

**Table 4:** MM-GBSA binding free energy results (kcal/mol).

Energy (kcal/mol)	Wild-Type EVC2/EVC (Mean ± SD)	Mutant EVC2/EVC (Mean ± SD)	*p* Value
ΔG_vdw_	46,467.8 ± 7.0	41,592.0 ± 26.8	<0.01
ΔG_ele_	−81,154.6 ± 31.5	−71,398.6 ± 36.1	<0.01
ΔG_polar_	−26,876.0 ± 43.6	−27,205.3 ± 56.1	<0.01
ΔG_nonpolar_	674.8 ± 0.7	600.5 ± 0.7	<0.01
ΔG_total_	−60,888.0 ± 14.5	−56,411.4 ± 19.8	<0.01

**Table 5:** Summary of clinical manifestations in patients with *EVC2* variants and tooth agenesis.

Variants	Location	Variant Type	Clinical Features	References
c.198_199insGGCGG	Exon1	Homozygous	atrial septal defect, short limbs, genu valgum, postaxial polydactyly, multiple oral frenulae, oligodontia, teeth dysplasia	[21]
c.1472C>T	Exon11	Heterozygous	oligodontia	[17]
c.1772C>T	Exon12	Homozygous	sparse hair, dry skin, prominent ears, oligodontia	[16]
c.1855C>T	Exon12	Heterozygous	short stature, short limbs, short ribs, postaxial polydactyly, hypoplastic nails, multiple oral frenulae, oligodontia, teeth dysplasia, fusion of the hamate and capitate	[21]
c.2653C>T	Exon15	Compound heterozygous	short stature, short limbs, genu valgum, postaxial polydactyly, hypoplastic nails, teeth dysplasia, oligodontia	[18]
c.3793del	Exon22	Homozygous	polydactyly, hypoplastic nails, multiple oral frenulae, delayed teeth eruption, oligodontia	[6]
c.3797T>A	Exon22	Heterozygous	postaxial polydactyly, hypoplastic nails, multiple oral frenulae, teeth dysplasia, oligodontia	[19]
c.3797T>G	Exon22	Heterozygous	postaxial polydactyly, hypoplastic nails, teeth dysplasia, oligodontia, osteopenia, mental delay	[19]
c.3805G>T	Exon22	Heterozygous	postaxial polydactyly, hypoplastic nails, enamel hypoplasia, oligodontia	[20]

## Data Availability

The datasets presented in this study can be found in NCBI Sequence Read Archive with the accession number PRJNA1310029.

## Supplementary Materials

The following supporting information can be downloaded at: https://www.mdpi.com/article/10.3390/genes16111288/s1, Figure S1. Photographs of the proband’s hands (A), feet (B), and joints (C). No significant abnormalities are observed. Figure S2. Echocardiogram of the proband. (A) Cardiac structure and left ventricular function. (B) Right ventricular function. The findings indicate normal cardiac structure and function without significant abnormalities. Figure S3. Photographs of the father’s intraoral photographs (A–C), hands (D), feet (E), and joints (F). No significant abnormalities are observed. Figure S4. Photographs of the mather’s intraoral photographs (A–C), hands (D), feet (E), and joints (F). No significant abnormalities are observed.

## Abbreviations

The following abbreviations are used in this manuscript:

NSTA	Non-syndromic tooth agenesis
WES	Whole-exome sequencing
SMO	Smoothened
RT-qPCR	Reverse transcription quantitative PCR
Hh	Hedgehog
RMSD	Root-mean-square deviation
MM-GBSA	Molecular Mechanics-Generalized Born Surface Area
SD	Standard deviation
RMSF	Root-mean-square fluctuation
