# Supplementary material for: Identification of a Novel EVC2 Variant in a Family with Non-Syndromic Tooth Agenesis and Its Potential Functional Implications
Source: Genes (Basel). 2025 Oct 30;16(11):1288. doi: 10.3390/genes16111288 (PMC12652662; doi:10.3390/genes16111288)
Supplement: Supplementary file 1 [file genes-16-01288-s001.zip › genes-3929346-supplementary.docx]

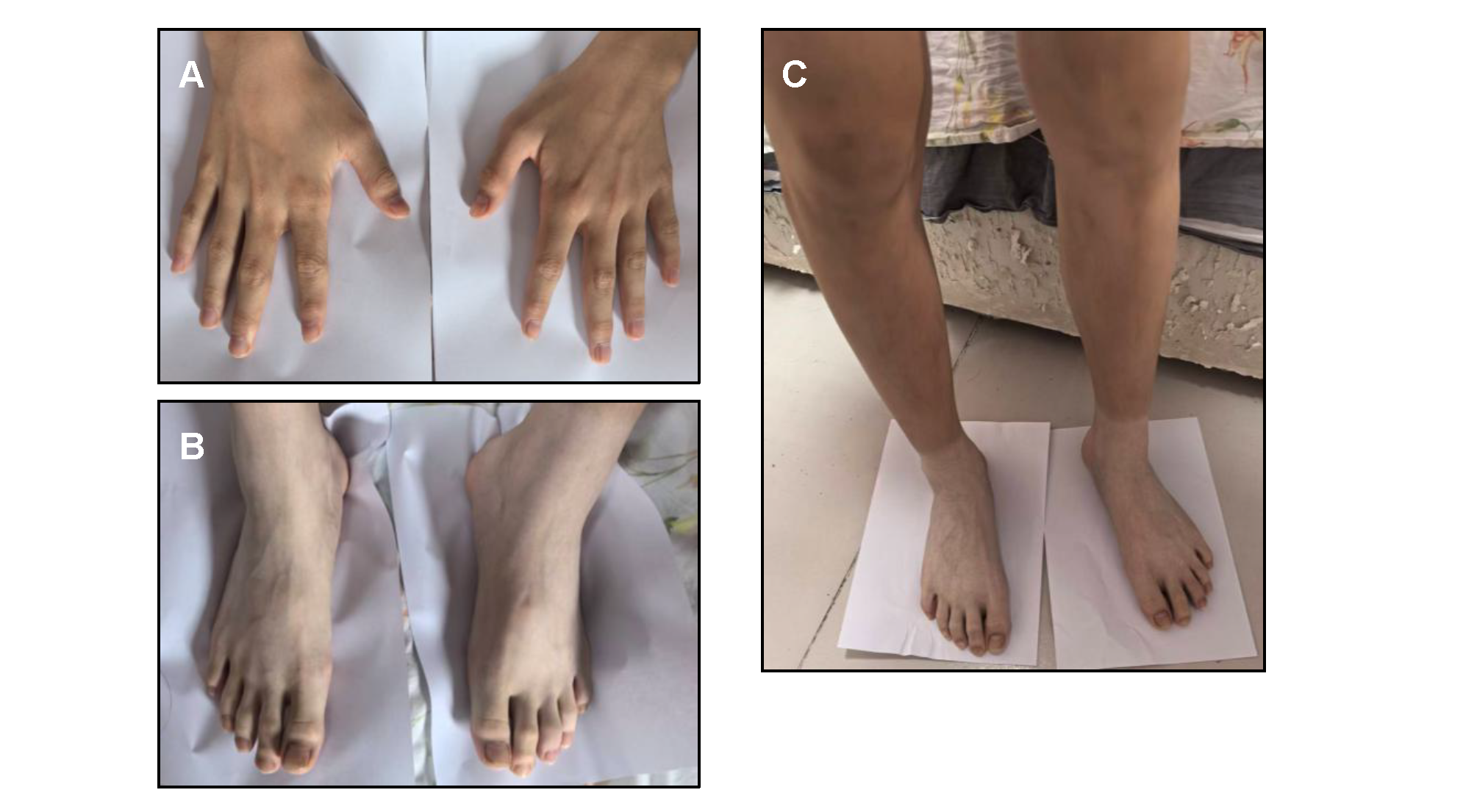


**Figure S1. Photographs of the proband's hands (A), feet (B), and joints (C).** No significant abnormalities are observed.


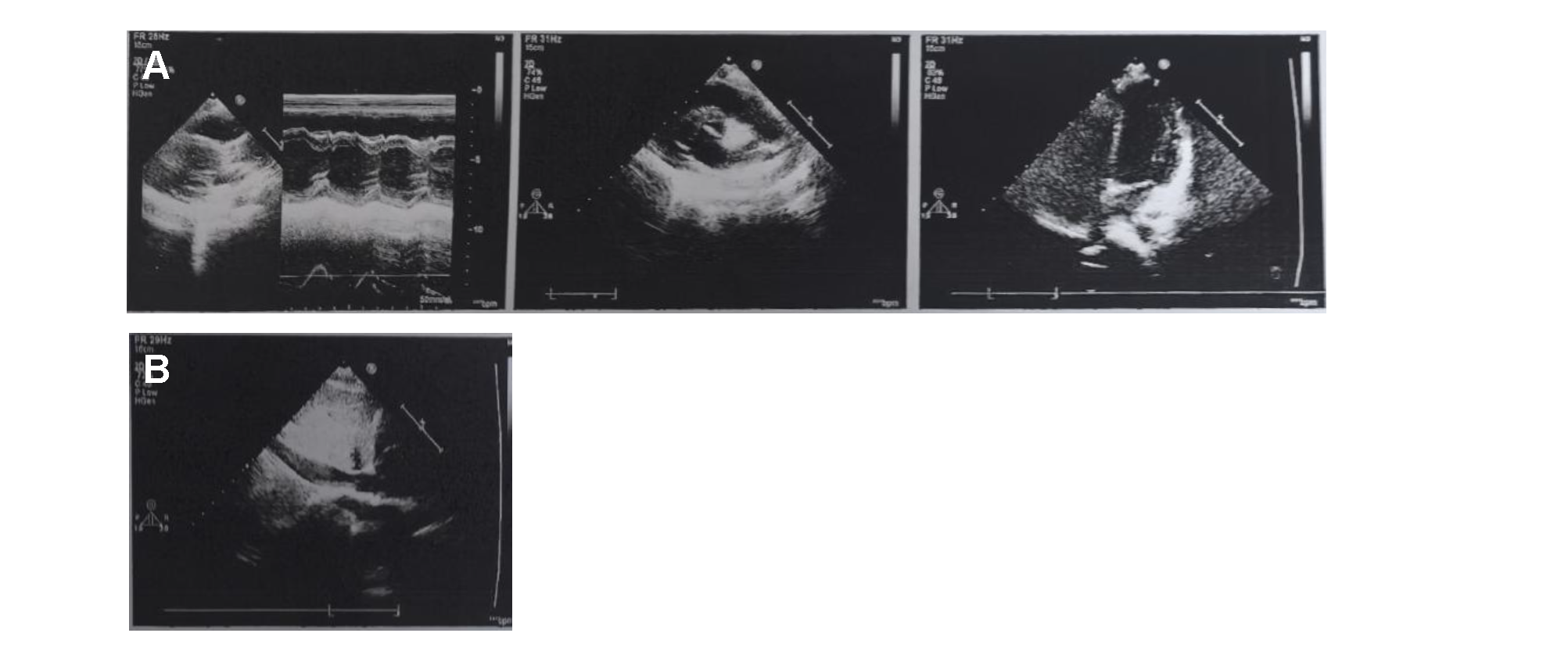


**Figure S2. Echocardiogram of the proband.** (**A**) Cardiac structure and left ventricular function. (**B**) Right ventricular function. The findings indicate normal cardiac structure and function without significant abnormalities.


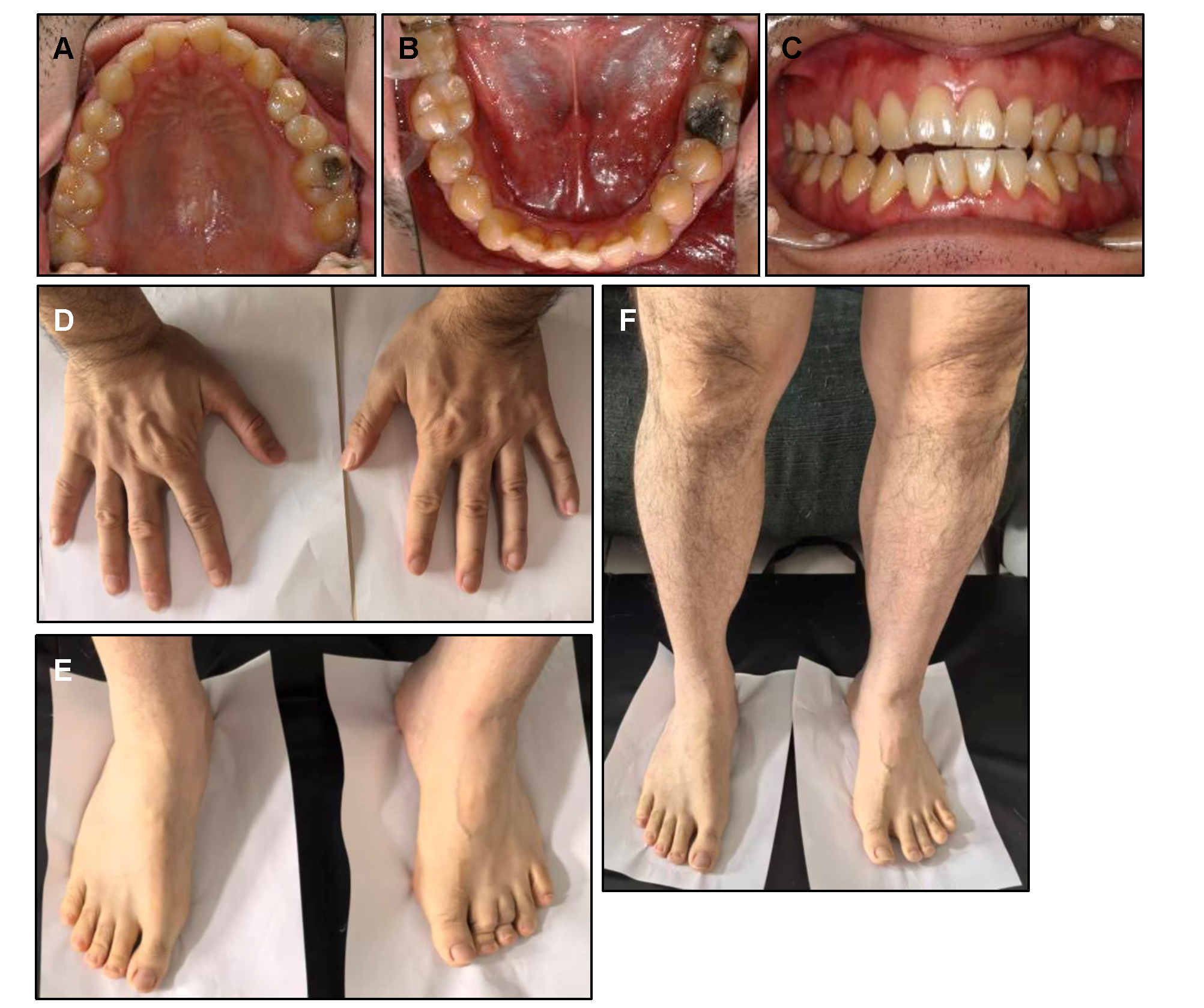


**Figure S3. Photographs of the father's intraoral photographs (A-C), hands (D), feet (E), and joints (F).** No significant abnormalities are observed.


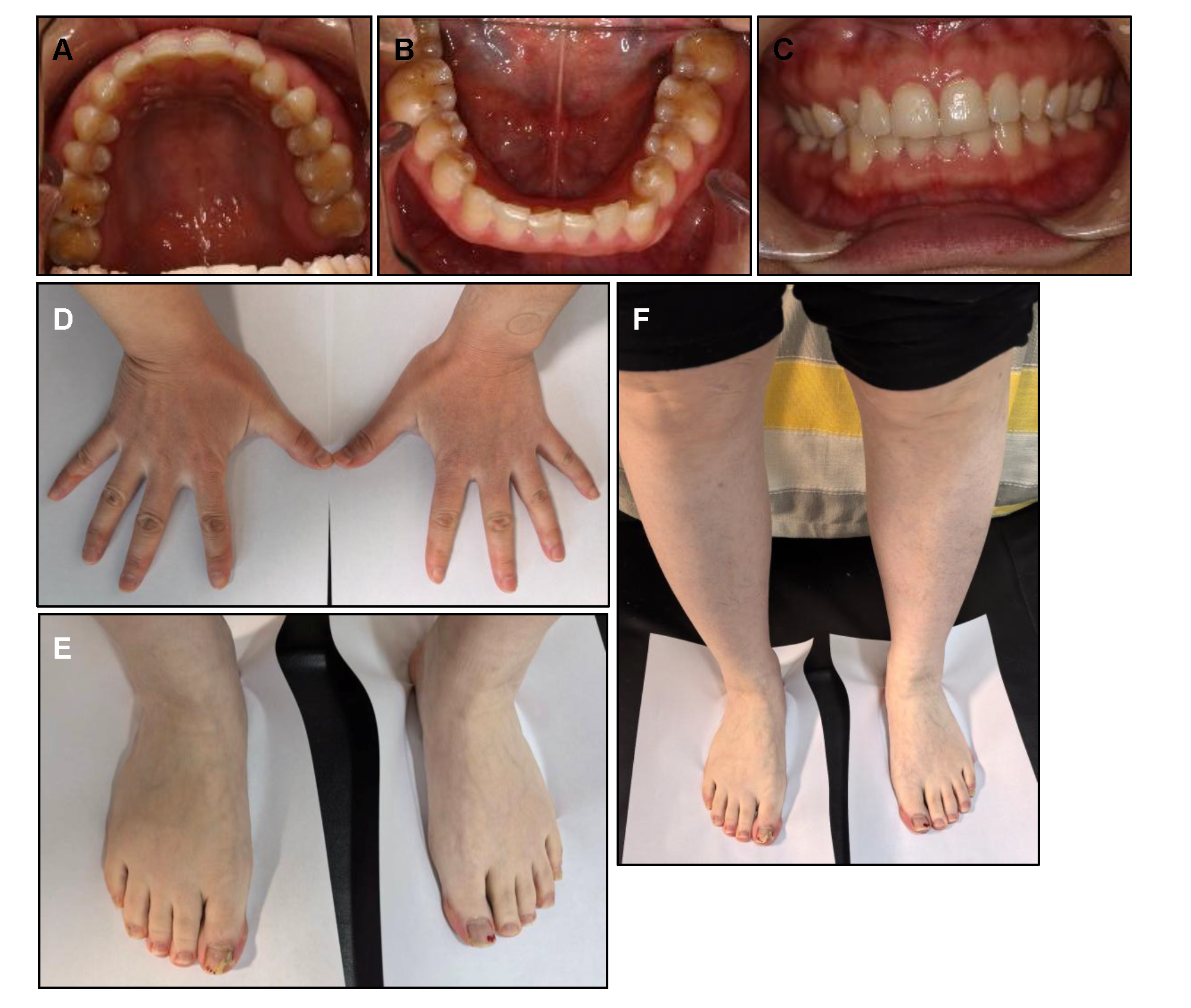


**Figure S4. Photographs of the mather's intraoral photographs (A-C), hands (D), feet (E), and joints (F).** No significant abnormalities are observed.
